# Acyl-Carrier-Protein-Engineering Rewires the Fatty Acid Profile in the Oleaginous Yeast *Rhodotorula toruloides*

**DOI:** 10.3390/jof12100750

**Published:** 2026-10-07

**Authors:** Shuang Wang, Qiongqiong Chen, Liting Lyu, Zongbao Kent Zhao

**Affiliations:** 1 Laboratory of Biotechnology, Dalian Institute of Chemical Physics, Dalian 116023, China; wangs@dicp.ac.cn (S.W.); chenqiongqiong@dicp.ac.cn (Q.C.); 2 University of Chinese Academy of Sciences, Beijing 100049, China; 3 MOE Key Laboratory of Bio-Intelligent Manufacturing, School of Bioengineering, Dalian University of Technology, Dalian 116024, China; lyult@dlut.edu.cn

**Keywords:** *Rhodotorula toruloides*, fatty acid synthase, acyl carrier protein, thioesterase, medium-chain fatty acids, fatty acid compositional profiles

## Abstract

The oleaginous yeast *Rhodotorula toruloides* has emerged as a host for the production of lipids mainly consisting of long-chain fatty acid triacylglycerides as alternative feedstock for oleochemicals and biofuels. The *R. toruloides* fatty acid synthase (*Rt*FAS) complex contains two acyl carrier protein (ACP) domains within its Fas2 subunit; it remains unclear whether inactivation of one ACP domain can lead to any biological effects, especially for fatty acid biosynthesis. Here, we constructed *R. toruloides* strains in which either one of the ACP domains was inactivated, and found that there was little difference in terms of cell growth and overall lipid production, suggesting these ACPs are functionally redundant. However, lipids from those strains contained lower contents of palmitic acid (C16:0). When one ACP was replaced with a truncated *Escherichia coli* thioesterase (‘TesA), known for the premature termination of the chain elongation process of fatty acid synthesis, these strains produced lipids with substantially higher contents of medium-chain fatty acids, corresponding to 6.3-fold and 4.2-fold increases in the relative abundance of C12:0 and C14:0, respectively, compared with the wild-type strain. This work shows that engineering the ACP domain of *Rt*FAS can be explored as a new strategy for modulating fatty acid compositional profiles in oleaginous yeasts.

## 1. Introduction

The oleaginous yeast *Rhodotorula toruloides* exhibits exceptional potential for natural carotenoid biosynthesis due to its native MVA pathway and lipid accumulation capacity [1,2,3,4]. It can produce lipids using a variety of low-cost carbon sources, with lipid accumulation reaching up to 70% of its dry cell weight under nitrogen-limited conditions [5]. Unlike the model yeast *Saccharomyces cerevisiae*, which belongs to the Ascomycota, *R. toruloides* is a basidiomycetous species with superior lipid accumulation capacity, higher stress tolerance, broader substrate scope, and robust growth under a wide range of industrially relevant conditions. [6,7]. These traits make it an ideal host for the scalable production of high-value oleochemicals. Although *R. toruloides* possesses a highly active fatty acid synthesis pathway, its lipid products consist mainly of C16 and C18 fatty acids (FAs), primarily the saturated FAs C16:0 and C18:0 and the monounsaturated FA C18:1. Recent metabolic engineering studies have engineered this yeast to produce a variety of high-value long-chain fatty-acid-derived lipids, including very-long-chain fatty acids (LCFAs) [8], long-chain polyunsaturated fatty acids [9], fatty alcohols [10,11], wax esters [12], and alkanes and alkenes [13], with reported titers ranging from hundreds of milligrams per liter to several grams per liter [14].

**Figure 1:**
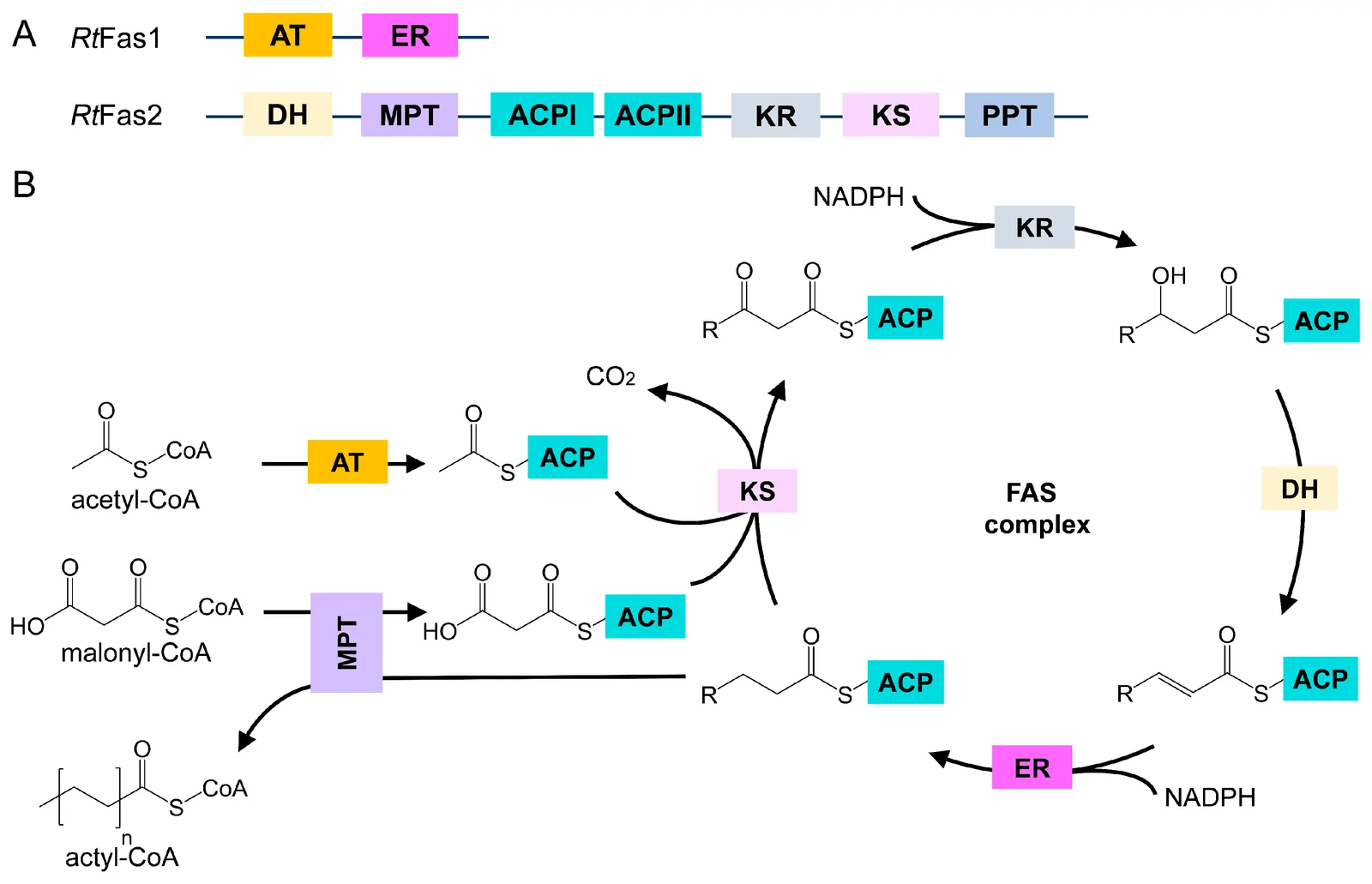
Architecture and reaction cycle of the fungal fatty acid synthase. (**A**) Schematic presentation of the native fungal FAS. (**B**) Reaction cycles catalyzed by fungal FAS: AT, acetyltransferase; ER, enoyl reductase; DH, dehydratase; MPT, malonyl/palmitoyl transferase; ACP, acyl carrier protein; KS, ketoacyl synthase; KR, ketoacyl reductase; PPT, phosphopantetheinyl transferase.

Among these fatty acids, lauric acid (C12:0) and myristic acid (C14:0) are of particular industrial importance owing to their broad applications in personal care formulations, pharmaceutical ingredients and specialty surfactants [15,16,17,18]. However, *R. toruloides* cannot efficiently accumulate medium-chain fatty acids under native conditions because its endogenous *Rt*FAS releases nascent acyl chains only after they are elongated to 16 or 18 carbons [19]. *Rt*FAS assembles into a 2.7 MDa heterohexameric α_6_β_6_ complex, a giant multifunctional molecular machine that catalyzes de novo fatty acid synthesis [20]. Encoded by the *FAS1* (β-subunit, Fas1) and *FAS2* (α-subunit, Fas2) genes (Figure 1A) [19], the complex operates through an iterative condensation cycle. In *Rt*FAS, there are two copies of the acyl carrier protein (ACP) domain, namely ACPI and ACPII. These two domains share 77% sequence identity and both have a dedicated serine residue that is responsible for charging fatty acyl chains [20]. Crucially, the termination of this cycle and the release of the fatty acid are governed by the malonyl/palmitoyl transferase (MPT) domain. The native MPT domain acts as a strict molecular ruler, harboring a stringent substrate preference that only liberates acyl chains after seven or eight elongation cycles, resulting exclusively in C16:0 and C18:0 FAs (Figure 1B) [20,21,22]. However, wild-type *R. toruloides* accumulates only low levels of medium-chain fatty acids (MCFAs). To address this limitation, we engineered one of the ACP domains of *Rt*FAS to reprogram chain termination and shift the fatty acid profile toward MCFAs.

In this study, we first confirmed that the inactivation of either ACP domain of *Rt*FAS in *R. toruloides* does not impair cell growth or lipid accumulation. Then, we swapped either one of the two ACP domains with the truncated thioesterase (‘TesA) from *Escherichia coli*, a well-characterized acyl-ACP thioesterase capable of hydrolyzing acyl intermediates of varying chain lengths [23,24,25], introducing an engineered chain-termination module into the endogenous FAS complex. This substitution provided an alternative termination route that arrests acyl chain elongation at 12 and 14 carbons and shifts fatty acid synthesis toward medium-chain fatty acids without reducing lipid content. The overall goal of this work is to establish ACP domain-swapping of *Rt*FAS as a viable strategy for modulating fatty acid compositional profiles in oleaginous yeasts.

## 2. Materials and Methods

### 2.1. Strains, Plasmids and Medium

Supplementary Tables S1 and S2 list the strains and plasmids used in the current study. *R. toruloides* NP11 was a haploid of CGMCC 2.1389 isolated by our lab, which is available from Guangdong Microbial Culture Collection Center (No. GDMCC 2.224).

All yeast strains were cultured in YPD medium (glucose 20 g/L, yeast extract 10 g/L, and peptone 20 g/L, native pH at 30 °C. *E. coli* DH5α and *E. coli* Fast-T1 were cultured using Luria–Bertani medium (LB, native pH) (NaCl 10 g/L, yeast extract 5 g/L, and tryptone 10 g/L) at 37 °C. *Agrobacterium tumefaciens* AGL1 was employed for *Agrobacterium*-mediated transformation (ATMT) of *R. toruloides*. The induction medium (IM) agar plates were prepared for ATMT. The Minimal Medium (MM) salt solution was composed of K_2_HPO_4_ 2.05 g/L, KH_2_PO_4_ 1.45 g/L, NaCl 0.15 g/L, MgSO_4_ 0.5 g/L, CaCl_2_ 0.006 g/L, FeSO_4_·7H_2_O 0.00248 g/L, (NH_4_)_2_SO_4_ 0.5 g/L. A total of 1 L of IM contained a 400 mL MM salt solution, 5 mL of glycerol, 15 g/L of agar, and 50 mM of 2-(N-morpholino) ethanesulfonic acid (MES). The pH was adjusted to 5.3 with 1 M HCl before autoclaving, and then filter-sterilized acetosyringone (AS) was added to a final concentration of 200 μM [26]. *A. tumefaciens* was cultured at 30 °C in LB medium. The yeast fatty acid auxotrophy of the *FAS2* gene deletion mutant was verified by growth on synthetic complete (SD) medium containing 6.7 g/L of the yeast nitrogen base without amino acids (YNB), 20 g/L of glucose, and 20 g/L of agar for the SD plate. For the culture of fatty acid auxotrophic strains, fatty acids (100× stock solution consisting of 50 mM of stearic acid and 50 mM of palmitic acid dissolved in a mixture of Tween 80 and ethanol (1:1, *v*/*v*)) were supplemented to the SD medium (native pH) [27]. The antibiotics employed in this study were kanamycin (50 μg/mL) and cefotaxime (300 μg/mL). For yeast fermentation, the engineered strains were cultured in nitrogen-limited (NL) medium (glucose 50 g/L, yeast extract 0.53 g/L, (NH_4_)_2_SO_4_ 0.4 g/L, MgSO_4_ 1.5 g/L, KH_2_PO_4_ 1.0 g/L, and 50 mM of MES). The pH was adjusted to 6.0 with 1 M NaOH before autoclaving, and the C/N ratio of the NL medium was 275:1 (mol/mol), calculated from the carbon content of glucose and the nitrogen content of (NH_4_)_2_SO_4_.

### 2.2. Reagents

All other chemicals were purchased from Bonuo Biological and Chemical Reagent Co., Ltd. (Dalian, China). All DNA polymerases and other restriction enzymes were acquired from Takara (Dalian, China). Kanamycin and acetosyringone were acquired from Dingguo Biotech Co., Ltd. (Beijing, China). The peptone, tryptone, and yeast extracts were produced by Oxoid (Basingstoke, UK). All of the primers used (Supplementary Table S3) were manufactured by Synbio Tech (Suzhou, China).

### 2.3. Plasmid Construction

The *‘tesA* gene-encoding thioesterase (GenBank accession: QXV67745.1, truncated *tesA* from *E. coli*) was optimized according to the codon preference of *R. toruloides* and synthesized by Synbio Technologies (Suzhou, China). The endogenous *FAS2* gene encodes the α-subunit from our lab [28]. To obtain the *FAS2*-knockout strain, the rational sgRNA corresponding to the particular locus of the *FAS2* gene (locus tag: RHTO_02139) was designed according to a previously described method [29]. The resulting fragments were inserted into plasmid pZPK. All plasmids were constructed using restriction-free cloning [30]. All target genes were inserted into the binary vector pZPK for ATMT-mediated transformation of *R. toruloides*. Each functional expression cassette was driven by the constitutive P*_GPD_* promoter and terminated by T*_HSP_*. All the primers used in this study are listed in Supplementary Table S3.

### 2.4. R. toruloides Transformation and Custruction

Transformation of *R. toruloides* was based on the ATMT methods [26]. Briefly, recombinant plasmids were introduced into competent *A. tumefaciens* cells via electroporation using an Eporator electroporation system (Eppendorf AG, Hamburg, Germany) with 0.1 cm gap cuvettes under parameters of 2.5 kV. Immediately after electric pulse treatment, 950 µL of LB medium was added for cell recovery at 30 °C for 1 h. Afterwards, 50 µL of bacterial suspension was spread onto LB agar medium containing 50 µg/mL of kanamycin. Positive *A. tumefaciens* transformants were cultured in kanamycin-supplemented LB medium (50 µg/mL) at 30 °C for 16 h. Meanwhile, *R. toruloides* cells were cultured in YPD medium at 30 °C for 16 h.

For strain construction, the *fas2Δ* mutant was generated by CRISPR/Cas9-mediated disruption of *FAS2* in NP11-SaCas9-1 (*Rt*WT) [29]. The full-length *FAS2* gene (8787 bp) was amplified from plasmid pET24b-FAS2 [28] and integrated into the *fas2Δ* mutant via ATMT [26], yielding strain *Rt*FAS2. In *Rt*FAS2, *FAS2* expression was driven by the GPD promoter to maintain constitutive expression of the Fas2 subunit with native ACPI and ACPII domains. The key Ser residue for each ACP domain was identified previously [27]. Single serine-to-alanine substitutions (S1026A and S1253A) were introduced by site-directed mutagenesis using pZPK-FAS2 as the template to generate mACPI and mACPII, respectively. Each mutant cassette was separately integrated into the *fas2Δ* background to obtain strains *Rt*FAS201, *Rt*FAS202, *Rt*FAS203, and *Rt*FAS204. Strains, plasmids, and primers are listed in Supplementary Tables S1–S3.

Both bacterial and fungal pellets were harvested by centrifugation at 13,000× *g* for 30 s, washed once, and adjusted to an OD_600_ of 0.6 with sterile distilled water. For co-cultivation, equal volumes (100 µL each) of the two cell suspensions were mixed and dropped onto IM agar plates supplemented with 200 µM of AS, followed by incubation at 25 °C for 48 h. The co-cultivation mixtures were collected and spread onto YPD agar supplemented with 300 µg/mL of cefotaxime to eliminate *A. tumefaciens* cells. Plates were incubated at 30 °C, and single colonies emerged within 2 days.

Primary transformants were picked, resuspended in sterile water, serially diluted, and spread on selective YPD plates for secondary screening to obtain pure monoclonal strains for genotypic identification. The purified strains were cultured in YPD medium, and OD_600_ values were detected every 40 min. The maximum specific growth rate (μ_max_) was calculated following a previously described method [31].

### 2.5. Shaking Flask Cultures

Seed cultures of *R. toruloides* were initiated by transferring a single colony from fresh YPD agar plates into 50 mL of YPD medium in a 250 mL Erlenmeyer flask, followed by incubation for 24 h. For lipid biosynthesis fermentation, seed suspensions were inoculated into 50 mL of NL medium. All culture groups were adjusted to an initial OD_600_ of 1.0 and incubated at 30 °C with shaking at 200 rpm. Samples were collected every 24 h to quantify cell density and residual glucose concentrations.

### 2.6. Determination of Dry Cell Weight and Total Carotenoid Content

A total of 1 mL of culture broth was harvested via centrifugation at 8000× *g* for 5 min. The cell pellet was washed twice with 1 mL of deionized water to remove residual medium components and salts. The washed biomass was transferred to a pre-weighed aluminum dish, dried at 105 °C for 12 h until constant weight, cooled in a desiccator for 2 h to room temperature, and weighed on an analytical balance. Dry cell weight (DCW) was calculated from the weight difference and expressed as grams per liter (g/L) of culture. All measurements were performed in triplicate, and mean values with standard deviations are reported.

For total carotenoid extraction, all operations were performed under dim light to prevent photodegradation. Briefly, 1 mL of culture broth was mixed with 300 μL of 3 M HCl and incubated in a boiling water bath for 3 min, followed by cooling in ice water for 3 min. The sample was centrifuged at 6200× *g* for 5 min, and the supernatant was discarded. The pellet was extracted three times with 300 μL of acetone each time until the cell pellet turned white, and the acetone supernatants were pooled into a single tube. To the combined acetone extract, 500 μL of petroleum ether and 300 μL of 0.1% (*w*/*v*) NaCl solution were added to facilitate phase separation. Liquid–liquid extraction with fresh petroleum ether was performed to transfer carotenoids into the organic phase. All petroleum ether extracts were combined and adjusted to a final volume of exactly 1.0 mL with petroleum ether. The absorbance of the solution was measured at 450 nm using a UV spectrophotometer. Total carotenoid content was calculated as micrograms per gram of dry biomass (μg/g DW) [32].

### 2.7. Lipid Extraction, Quantification, and Fatty Acid Compositional Analysis

At the end of fermentation, 30 mL of broth was sampled and centrifuged at 8000× *g* for 5 min to harvest yeast cells. The cell pellet was washed twice with 40 mL of deionized water to remove residual medium components and salts. The resulting pellets were dried at 105 °C for 12 h to constant weight, equilibrated in a desiccator for 2 h to room temperature, and weighed to determine DCW. The cell mass was measured gravimetrically and expressed in g/L (total dry cells produced per liter). To extract the lipid, the dried cells were first digested in 4 M HCl at 78 °C for 1 h with 200 rpm shaking, and then extracted with 4 mL methanol and 4 mL chloroform. After thorough vortexing and centrifugation at 8000× *g* for 5 min at room temperature, the lower chloroform layer was transferred to a clean 50 mL centrifuge tube. The remaining aqueous phase was re-extracted with 4 mL of fresh chloroform by repeating the same vortexing and centrifugation steps until the chloroform layer became colorless. All chloroform extracts were combined and washed with an equal volume of 0.1% (*w*/*v*) NaCl solution, then vortexed and centrifuged at 8000× *g* for 5 min. The purified chloroform phase was collected, and the solvent was removed by rotary evaporation under reduced pressure. The concentrated residues were dried at a constant weight at 105 °C for 12 h to constant weight, equilibrated in a desiccator for 2 h to room temperature, and weighed to determine lipids gravimetrically. The total lipid produced was indicated as lipid titer and expressed in g/L, whereas the cellular lipid content was expressed as g lipid/g DCW (%).

For total fatty acid compositional analysis, fatty acid methyl ester (FAME) analysis was performed as follows. A total of 1 mL of fermentation broth was collected by centrifugation at 8000× *g* for 5 min and washed once with deionized water. Then, the washed cells were taken into glass vials, and 2 mL of 5% (*v*/*v*) sulfuric acid/methanol solution was added. The mixture was incubated at 85 °C for 90 min and then cooled to room temperature. A total of 200 μL of n-hexane and 500 μL of 0.9% (*w*/*v*) NaCl aqueous solution were added to extract the reaction products [33,34]. Finally, the mixture was vortexed for 10 min and centrifuged at 3000× *g* for 5 min. The hexane phase was collected for analysis by gas chromatography-mass spectrometry (GC-MS). The procedure was adapted from a previously reported method [2]. Fatty acid chromatographic peak areas were normalized by total area to calculate the relative abundance of individual species, expressed as percentages of total fatty acids (%TFA). Using these normalized values, two partitioning indices, (C12:0 + C14:0)/C16:0 and C16/C18, were defined in this study and computed to characterize *Rt*FAS-dependent chain-termination and post-*Rt*FAS elongation profiles in this study.

## 3. Results

### 3.1. Validation of the Biological Function of Individual ACP

**Figure 2:**
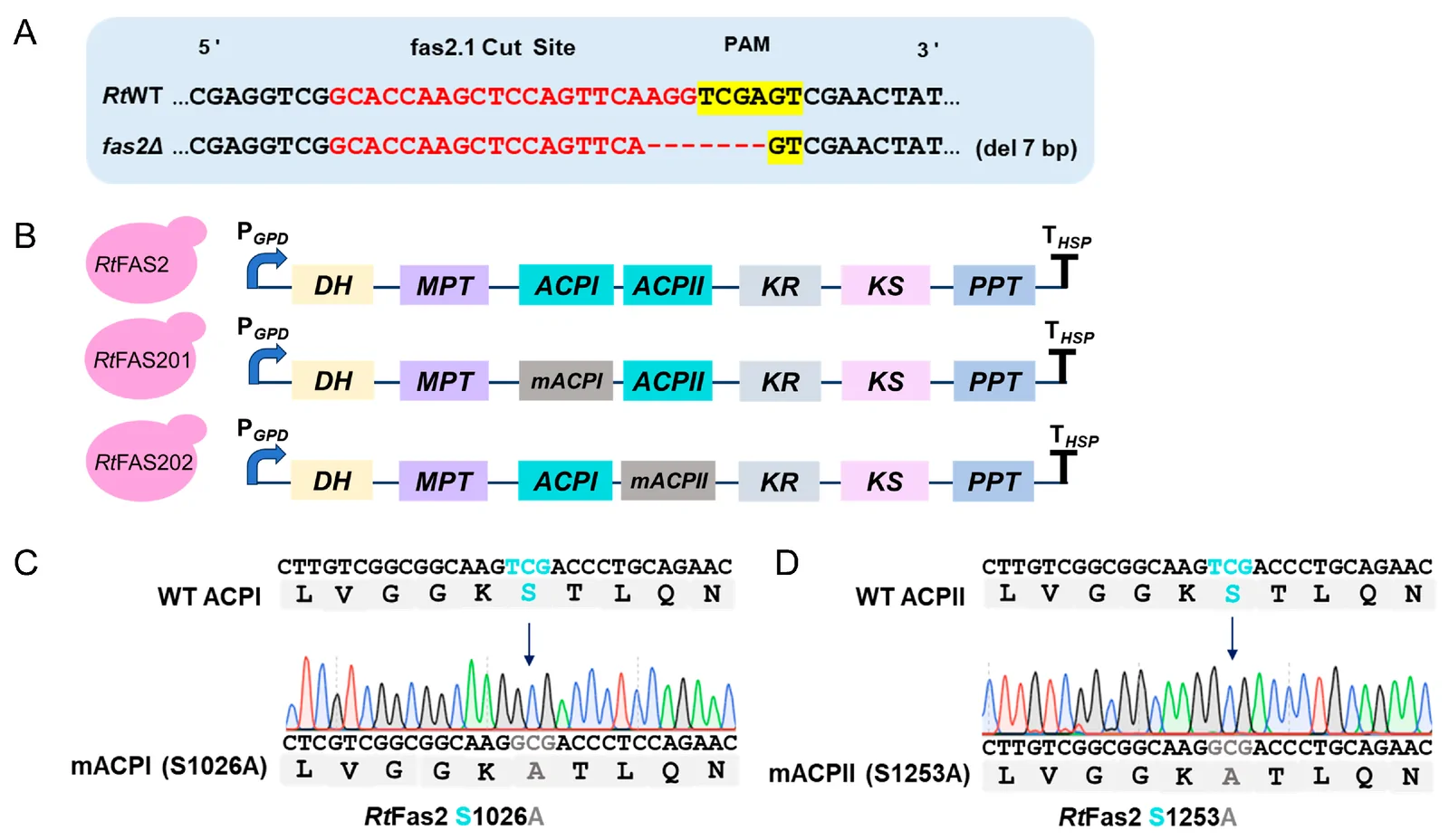
Validation of the biological function of the individual ACP domain in *R. toruloides*. (**A**) Sequence alignment shows a 7-base pair (7 bp) deletion at the SaCas9 cleavage site in the *fas2Δ* mutant compared with the *Rt*WT strain. (**B**) Schematic diagram of the expression cassettes integrated into the genome of the *fas2Δ* mutant of *R. toruloides*. The conserved serine residues of ACPs were substituted with alanine residues to inactivate the ACP domains (mACPI and mACPII). (**C**) Sequencing verification of site-directed mutagenesis for ACPI domain inactivation strain *Rt*FAS201. (**D**) Sequencing verification of site-directed mutagenesis for ACPII domain inactivation strain *Rt*FAS202.

To evaluate whether the inactivation of one ACP domain in *Rt*Fas2 affects fatty acid synthesis, we constructed a set of engineered *R. toruloides* strains. Disruption of the *FAS2* gene generated the *fas2Δ* mutant (Figure 2A and Figure S1), which failed to grow on SD medium but grew on SD medium supplemented with exogenous fatty acids (Figure S2), indicating a complete loss of de novo fatty acid synthesis. To restore fatty acid synthesis, the full-length *FAS2* gene was integrated into the *fas2Δ* mutant, yielding strain *Rt*FAS2 (Figure 2B and Figure S3), which harbors both native ACPI and ACPII domains. We then introduced single serine-to-alanine substitutions into each ACP domain and integrated the mutant cassettes into the *fas2Δ* background, yielding strains *Rt*FAS201 and *Rt*FAS202 (Figure 2B). Detailed construction procedures are described in Materials and Methods. The colonies on YPD agar plates showed that all engineered strains closely resembled the parental strain *Rt*FAS2 (Figures S2 and S3). Both *Rt*FAS201 and *Rt*FAS202 were genotyped by diagnostic PCR (Figure S4). The PCR products were subjected to analysis, confirming the precise serine-to-alanine substitution at each target locus (Figure 2C,D), thereby verifying the successful construction of the designed strains. These results demonstrated that inactivation either ACP domain did not adversely affect the basal growth capacity of *R. toruloides*. In addition, we evaluated the growth characteristics of each strain in YPD medium. The maximum specific growth rate and the total fatty acid composition of strains at the logarithmic growth phase are provided in Supplementary Table S4 and Figure S5, respectively. No significant differences in growth capacity were observed across all strains. Collectively, the loss of one ACP activity neither impairs cell growth nor perturbs the fatty acid compositional profiles. Loss of function of one ACP can be fully compensated by the other, demonstrating functional redundancy between the two ACPs and showing that a single ACP domain can support normal growth and de novo fatty acid synthesis in *R. toruloides*.

### 3.2. Effect of ACP Inactivation on Lipid Production

**Figure 3:**
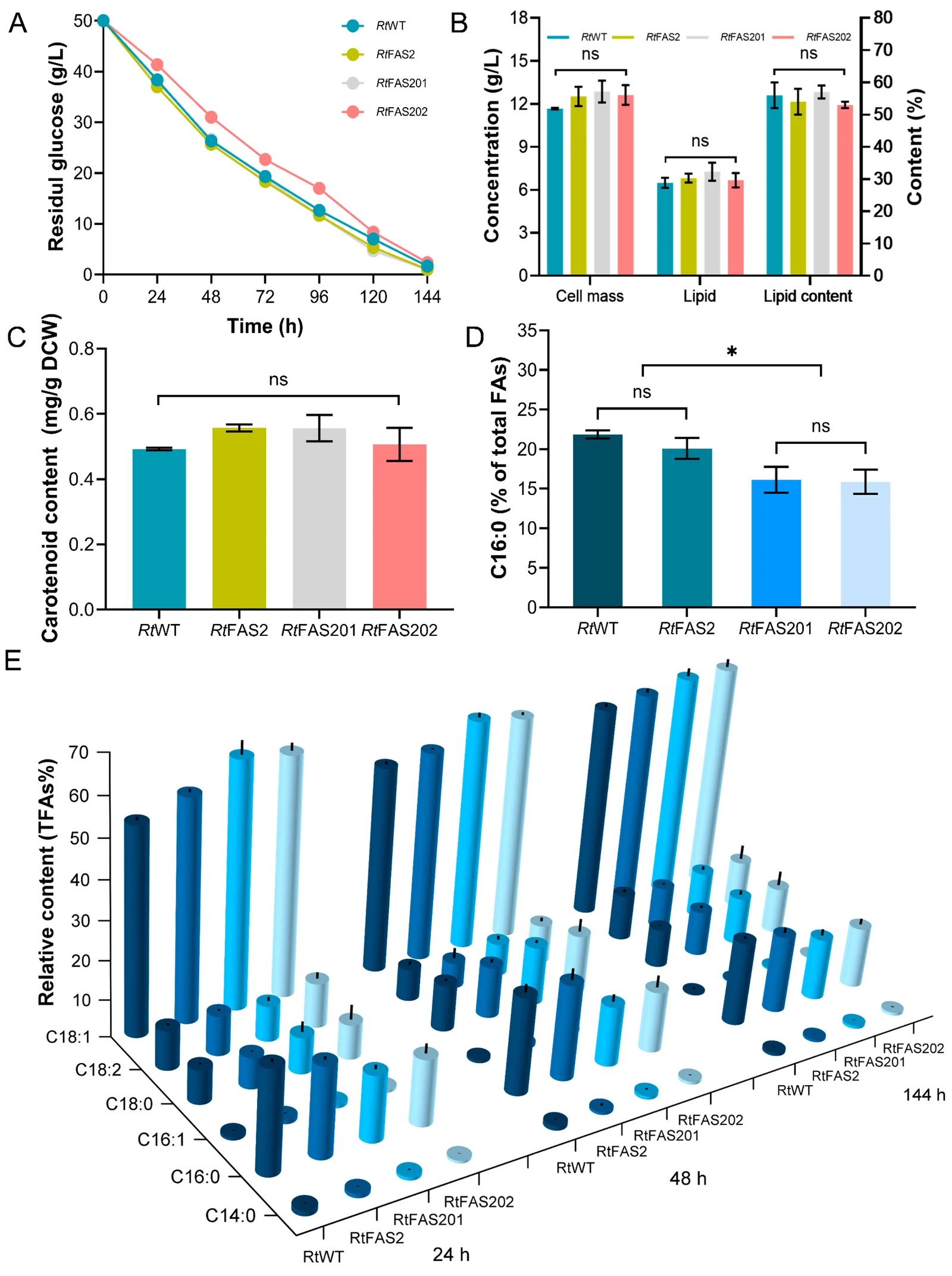
Lipid production with 50 mL culture in 250 mL shaking flasks by *R. toruloides* strains. (**A**) Residual glucose consumption curves of *Rt*WT, *Rt*FAS2, *Rt*FAS201, and *Rt*FAS202 during fermentation. (**B**) Cell mass, lipid titer, and lipid content of all strains at 144 h. (**C**) Carotenoid content of the strains at 144 h. (**D**) Relative proportion of C16:0 fatty acid at 144 h. (**E**) Dynamic changes in fatty acid composition (relative content % of total fatty acids, TFAs) of the strains at 24 h, 48 h, and 144 h. ns, no significant difference. Data represent the mean ± SD, *n* = 3 (biological replicates). Statistical comparisons were performed using an unpaired, two-tailed *t*-test with Welch’s correction. ns, not significant. *p* > 0.05; * *p* < 0.05.

To evaluate the effect of single-ACP-domain modification on lipid accumulation, strains were cultivated in NL medium in 250-mL shake flasks, with samples harvested every 24 h to quantify cell density, residual glucose, and fatty acid composition. As shown in Figure 3, all ACP-engineered strains displayed profiles highly comparable to those of *Rt*WT. The time-course profiles of glucose consumption overlapped among all strains (Figure 3A). At the end, no significant differences in cell mass, lipid titer, or cellular lipid content were observed between mutant strains and the control *Rt*WT (Figure 3B). Likewise, carotenoid biosynthesis remained unperturbed across all genotypes (Figure 3C). Taken together, these findings demonstrated that modification of the ACP domain does not alter the innate metabolic capacity for cell growth and lipid biosynthesis in *R. toruloides*.

Following physiological characterization of cell growth, lipid synthesis, and carotenoid production, we further profiled six major fatty acids (C14:0, C16:0, C16:1, C18:0, C18:1, C18:2) across three time points (24, 48, and 144 h) of the culture. It was found that the relative proportion of C16:0 was consistently lower in mACP mutants (*Rt*FAS201 16.1 ± 1.6%, *Rt*FAS202 15.0 ± 1.5%) compared with control strains (*Rt*WT 21.9 ± 0.5%, *Rt*FAS2 20.0 ± 1.3%) at 144 h (Figure 3D). Genetic complementation in *Rt*FAS2 fully restored the C16:0 proportion to wild-type levels, that the reduction in C16:0 is directly linked to ACP domain sequence alteration. Attributed to ACP domain sequence alteration, endpoint quantification at 144 h further validated this sustained phenotypic difference. Excluding C16:0, no other fatty acid species exhibited consistent mutation-associated trends across the fermentation period (Figure 3E and Figure S6). Notably, control strains displayed gradual shifts in fatty acid composition, primarily reflected in time-dependent variation in C18:1. In contrast, mACP mutants maintained stable fatty acid profiles throughout cultivation. Collectively, these results demonstrated clear phenotypic decoupling between total lipid biosynthesis and fatty acid chain-length distribution: ACP-domain mutation remodels fatty acid compositional profiles without compromising overall lipid yields.

### 3.3. Targeted ‘TesA Integration Alters Fatty Acid Composition

**Figure 4:**
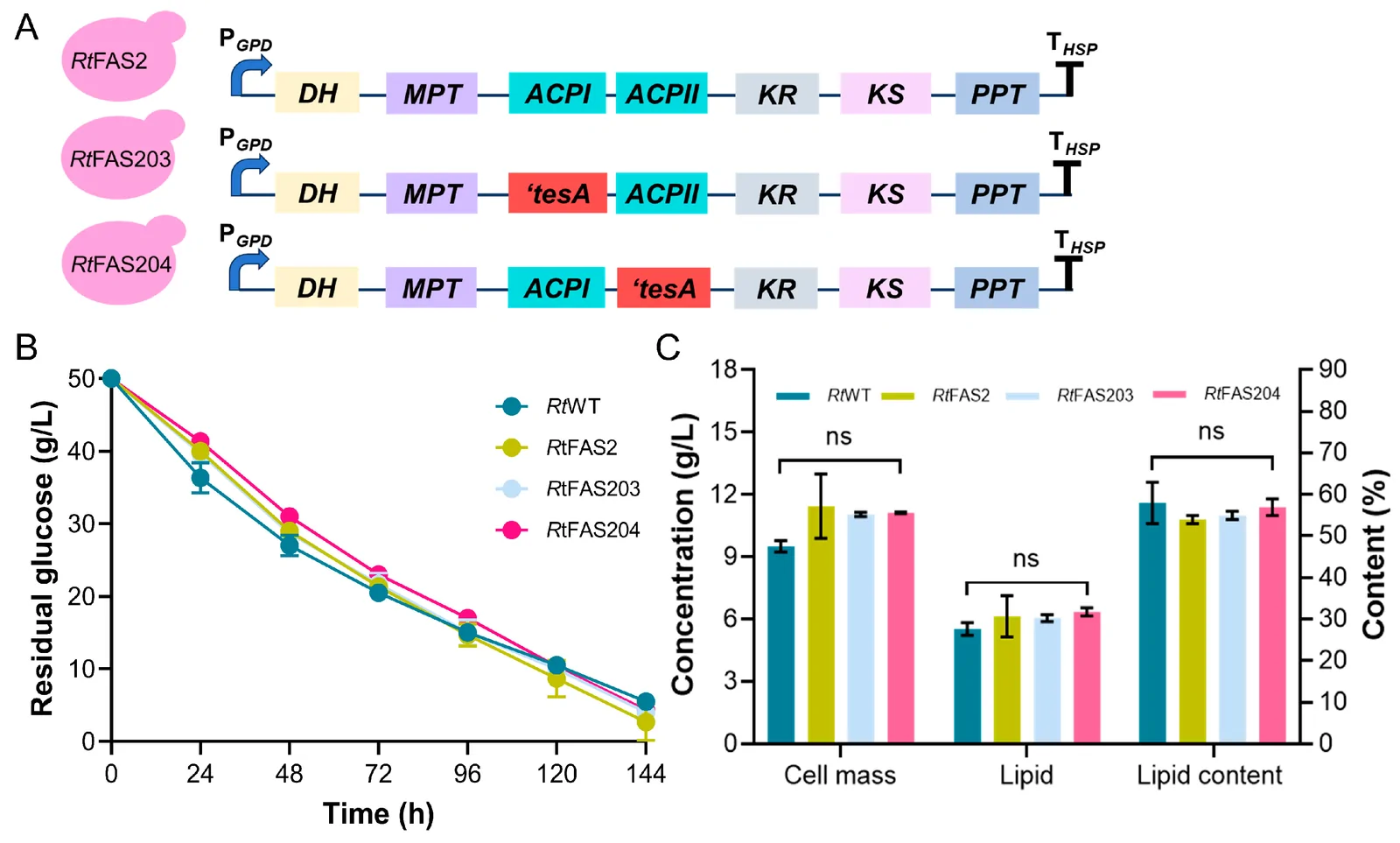
Site-directed ‘TesA integration and its effects on *R. toruloides* growth, lipid production, and myristic acid accumulation. (**A**) Schematic illustration of *Rt*Fas2 domains architecture in engineered strains: *Rt*FAS2 (wild-type *Rt*Fas2 control), *Rt*FAS203 (‘TesA replacing full ACPI domain), *Rt*FAS04 (‘TesA replacing full ACPII domain). (**B**) Residual glucose concentration over 144 h of fermentation. (**C**) Cell dry weight, lipid titer, and lipid content at endpoint (144 h). Data represent the mean ± SD, *n* = 3 (biological replicates). Statistical comparisons were performed using an unpaired, two-tailed *t*-test with Welch’s correction. ns, not significant. *p* > 0.05.

Building on our previous finding that ACP domain mutagenesis remodels fatty acid profiles without impairing lipid anabolism, we reprogrammed fatty acid chain length distribution in *R. toruloides* via domain swapping within *Rt*Fas2. To enrich the medium-chain fatty acids, the truncated *Escherichia coli* thioesterase (‘TesA) was used to replace either the ACPI or ACPII domain of *Rt*Fas2, generating the *Rt*FAS203 and *Rt*FAS204 strains, respectively (Figure 4A). The wild-type strain *Rt*WT and the strain *Rt*FAS2 were used as controls to disentangle the effect of ACP domain removal from ‘TesA-mediated hydrolytic activity. Strain construction and genotypic validation followed the workflow established for mACP mutants (Figures S1, S2 and S4).

To evaluate the lipid-producing performance, the engineered strains were tested in shake flasks under NL conditions. We took samples every 24 h for routine analysis. All strains exhibited comparable glucose consumption profiles. Residual glucose declined gradually over 144 h. *Rt*WT, *Rt*FAS2, *Rt*FAS203, and *Rt*FAS204 achieved comparable final cell mass, lipid titer, and lipid content at the endpoint (144 h) with no significant differences among groups (Figure 4C). These results show that ACP domain replacement with ‘TesA does not impair cell growth or lipid accumulation under the tested culture conditions.

**Figure 5:**
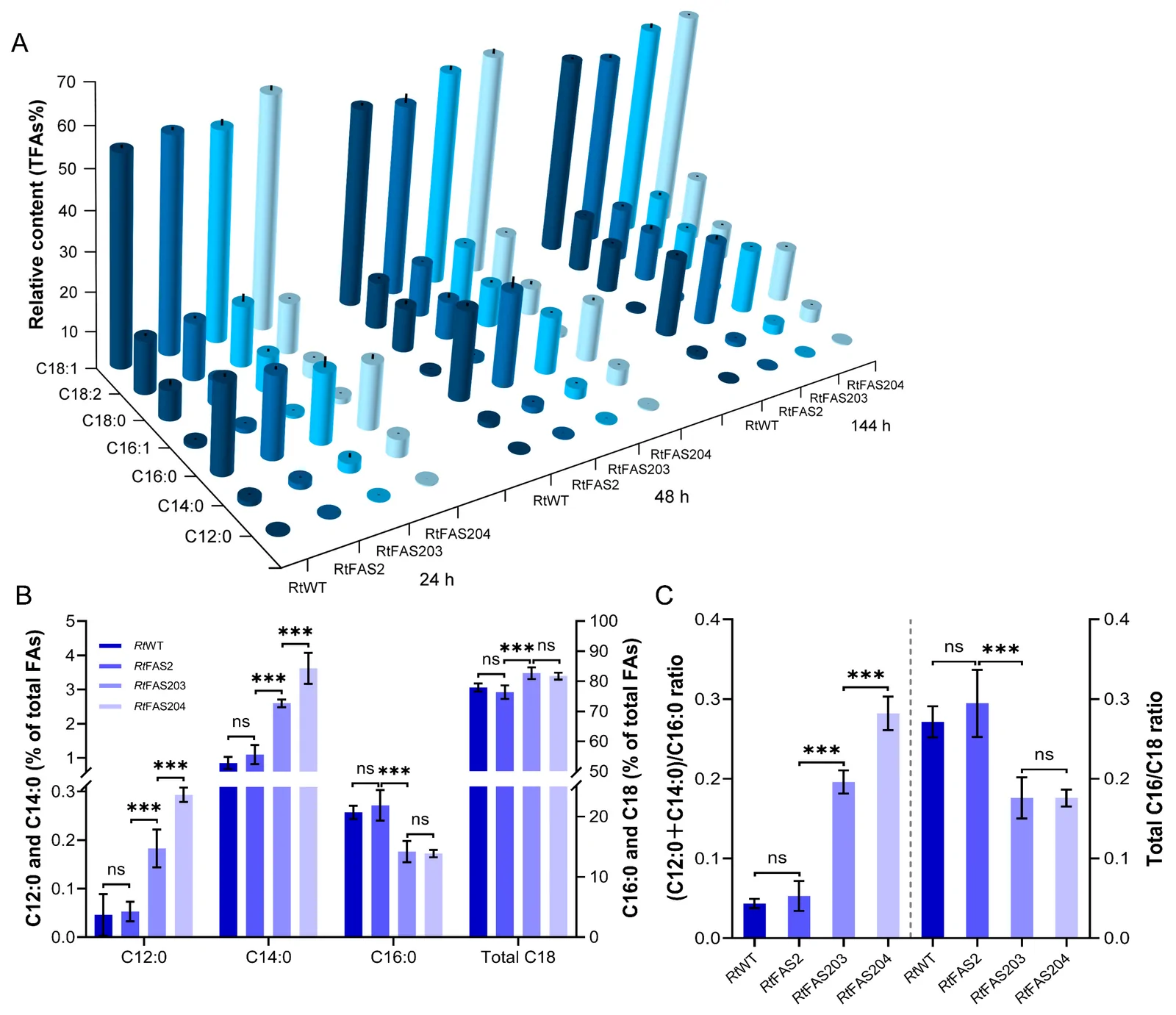
Fatty acid compositional profiling of the strains in NL medium. (**A**) Dynamic changes in fatty acid composition (relative percentage of total fatty acids) at 24 h, 48 h, and 144 h. Data are presented as mean ± SD, *n* = 3 (biological replicates). (**B**) Relative proportions of saturated fatty acids (C12:0, C14:0, and C16:0) were measured at the culture endpoint. (**C**) The C16/C18 ratio is calculated as the ratio of the total relative abundance of all C16-series fatty acids to that of all C18-series fatty acids, encompassing all saturated and unsaturated isoforms for each chain length at the culture endpoint. Strains: *Rt*WT, *Rt*FAS2, *Rt*FAS203, *Rt*FAS204. Statistical comparisons were performed using an unpaired, two-tailed *t*-test with Welch’s correction. ns, not significant. *p* > 0.05; *** *p* < 0.001.

Time-course analyses of fatty acid compositions were conducted at 24, 48, and 144 h to characterize the temporal dynamics of chain-length distributions across all strains (Figure 5A). *Rt*WT and the Fas2-complemented strain *Rt*FAS2 exhibited nearly superimposable profiles throughout cultivation, dominated by C16 and C18 fatty acids. This congruence verified that neither Fas2 complementation nor the ATMT procedure disrupts basal fatty acid homeostasis. In contrast, the engineered strains *Rt*FAS203 and *Rt*FAS204 displayed enrichment of the medium-chain acids C12:0 and C14:0 alongside a parallel reduction in the abundance of C16:0, which was detectable as early as 24 h and persisted throughout the lipid accumulation phase. At 144 h, the relative abundances of C12:0 and C14:0 in *Rt*FAS203 and *Rt*FAS204 were 3.9- and 3.0-fold and 6.3- and 4.2-fold higher than *Rt*WT, respectively (Figure 5B).

The relative abundance of monounsaturated C18:1 remained consistent across all strains, accounting for 50–60% of total fatty acids, and only a minor increase in polyunsaturated C18:2 was detected in the engineered strains, primarily at the late stage of the culture. Given that total lipid titers were comparable across all strains and that C18 fatty acids were not reduced, the elevated abundance of C12:0 and C14:0 reflects a net enrichment of these species rather than a passive compositional shift caused by suppressed fatty acid synthesis. This phenotype is consistent with the well-documented intrinsic substrate preference of the introduced ‘TesA for medium-chain acyl-ACP intermediates [24,25].

To further verify whether the observed chain-length redistribution is driven by ‘TesA-mediated premature hydrolysis of acyl-ACP intermediates within the *Rt*FAS, two partitioning indices were calculated based on endpoint fatty acid profiles (Figure 5C). The (C12:0 + C14:0)/C16:0 ratio was significantly higher in the engineered strains (0.19 for *Rt*FAS203, 0.28 for *Rt*FAS204) than in the control strains (0.04 for *Rt*WT, 0.05 for *Rt*FAS2). The C16/C18 ratio was 0.27 and 0.29 for *Rt*WT and *Rt*FAS2, respectively, and 0.17 and 0.18 for *Rt*FAS203 and *Rt*FAS204, respectively, representing a significant reduction in the engineered strains. A significant increase in the abundance of total C18 fatty acids was detected in the engineered strains. Together, these two indices indicated a significant redistribution of cellular fatty acid chain-length composition in the engineered strains, characterized by enrichment of C12:0 and C14:0 at the expense of C16:0.

In summary, integration of the truncated ‘TesA at the ACP domain loci of the *R. toruloides* type I FAS resulted in remodeling of the fatty acid profile and enrichment of C12:0 and C14:0, without compromising total lipid accumulation. The enrichment of medium-chain fatty acids across the cultivation period indicated that the chimeric FAS complexes remain functionally active.

## 4. Discussion

In this study, we performed domain-level engineering of the type I FAS from the oleaginous yeast *R. toruloides* and achieved targeted remodeling of the total fatty acid profile with specific enrichment of the medium-chain fatty acids C12 and C14. The results demonstrated that inactivating a single ACP did not impair cell growth or lipid accumulation, verifying the functional redundancy of the two ACP domains. Intriguingly, single ACP inactivation reduced the proportion of C16:0, consistent with the decreasing trend in C16:0 observed in single ACP mutants of *Rt*FAS expressed in a heterologous *S. cerevisiae* system ([27], Supplementary Table S2). However, the magnitude of change differed between hosts, likely attributable to the distinct native metabolic contexts of the two systems. Although engineered FAS2 variants were expressed under the strong constitutive GPD promoter for complementation. This aligns with the well-established architectural and assembly principles of fungal type I FAS [35,36,37]. Functional fungal FAS complexes require the coordinated expression and stoichiometric assembly of both Fas1 and Fas2 subunits, and overall fatty acid synthetic capacity is governed by the stoichiometric balance of the two subunits rather than the abundance or catalytic activity of either individual subunit. Building on the functional redundancy of the dual ACP domains, we adopted a domain-swapping strategy by replacing individual ACP domain with the truncated *E. coli* thioesterase ‘TesA. This strategy was designed to redirect the fatty acid profile toward medium-chain species while preserving the overall architecture and catalytic integrity of the endogenous FAS complex. No growth inhibition was detected in engineered strains, and total lipid accumulation remained at wild-type levels, demonstrating that domain-level modification is well tolerated by cellular metabolism and represents a gentle yet effective approach for targeted fatty acid profile engineering. Given the comparable total lipid titers across all strains and no depletion of total C18 fatty acids, the elevated relative abundance of C12:0 and C14:0 in engineered strains—under the condition of comparable total lipid titers—corresponds to a proportional increase in their amounts, indicating net enrichment of medium-chain species rather than a passive compositional shift. Notably, the total C18 proportion in the complemented strains (*Rt*FAS203/204) was significantly higher than in *Rt*WT and *Rt*FAS2. We attribute this not to enhanced elongation activity, but rather to a passive consequence of reduced C16 contribution: as carbon flux is preferentially diverted at the C12/C14 stage by the heterologous ‘TesA, less substrate enters the C16:0 pool, thereby increasing the relative share of C18 in the remaining long-chain fraction. This pattern further supported the substrate-length preference of ‘TesA for shorter acyl intermediates and demonstrated that the native FAS elongation machinery remains highly functional for converting C16 to C18.

Site-specific integration of ‘TesA at the two distinct ACP loci resulted in different degrees of C14:0 enrichment, forming a clear gradient of chain-length remodeling efficiency: *Rt*FAS204 consistently exhibited a stronger enrichment effect than *Rt*FAS203 across all fermentation time points. At the culture endpoint, the relative abundance of C12:0 and C14:0 in *Rt*FAS204 reached a 6.3- and 4.2-fold increase relative to the wild-type control *Rt*WT. This compositional shift is consistent with a model in which the integrated ‘TesA mediates premature hydrolysis of C14-acyl-ACP intermediates and redirects metabolic flux from saturated long-chain fatty acids toward medium-chain species. This domain-position-dependent regulatory pattern aligns with the core mechanistic principle of fatty acid chain-length control recently established in metazoan FAS systems: the final chain length is governed by the kinetic competition between KS-mediated acyl chain elongation and TE-mediated hydrolytic chain termination [25]. The site-dependent gradient observed in our study likely arises from differences in spatial proximity and interdomain interaction efficiency between the integrated ‘TesA and the endogenous KS domain at the two ACP loci, thereby modulating the relative strength of hydrolytic termination versus chain elongation.

Intriguingly, this functional divergence of tandem ACP domains exhibits notable parallels to the regulatory patterns observed in modular type I bacterial polyketide synthases (PKS). For instance, tandem ACP domains involved in mupirocin biosynthesis in *Pseudomonas fluorescens* exert functions beyond simple substrate carrier activity; their functional roles are shaped by interdomain interaction kinetics and spatial conformation with adjacent catalytic domains such as ketoreductase (KR). Dual ACPs can enhance synthetic flux through parallel intermolecular interactions, while triple ACPs achieve functional division via a combined tandem-parallel configuration [38]. Drawing on these shared regulatory principles and our in vivo phenotypic data, we proposed a working hypothesis that the functional differentiation of the dual ACP domains in *Rt*Fas2, as well as the graded chain-length remodeling efficiency across the two ‘TesA insertion sites, originates from divergent kinetic interaction parameters between individual ACP domains and their adjacent catalytic domains. While direct structural and biophysical validation of these domain-domain interactions is not provided in the current study, two consistent phenotypes, namely, the specific reduction in C16:0 upon single ACP inactivation and the site-dependent gradient of ‘TesA-mediated profile remodeling’, provide functional evidence in support of this hypothesis. Follow-up structural biology research and in vitro interaction assays could help validate the underlying molecular mechanism, laying a theoretical foundation for rational FAS engineering and efficient synthesis, as well as modification of MCFAs.

This work extended our understanding of tandem ACP regulatory patterns from bacterial PKS to eukaryotic type I FAS and identified a previously unreported role of dual ACP domains in modulating fatty acid chain length in fungal FAS. From a mechanistic perspective, our findings add a new layer of complexity to the chain-length regulation of fungal type I FAS and complement the established kinetic competition model of FAS chain-length control. From a metabolic engineering perspective, the ACP domain substitution strategy established here provided a mild and precise approach for tailoring the fatty acid profile of oleaginous yeasts. Unlike strategies that globally perturb fatty acid metabolism, this domain-level modification enables targeted redirection of carbon flux within the FAS complex without compromising overall lipid biosynthetic capacity. This approach expanded the metabolic engineering toolbox for FAS modification in oleaginous yeasts and offers a feasible technical route for constructing microbial cell factories with customized fatty acid composition profiles.

## 5. Conclusions

This work demonstrates that the two ACP domains of *Rt*Fas2 are functionally redundant for cell growth and overall lipid synthesis in *R. toruloides* but can regulate the content of C16:0 in total lipids. Moreover, the replacement of one ACP domain with ‘TesA enables efficient reprogramming of fatty acid chain length toward medium-chain species. These findings provide insights into FAS domain function and establish a new engineering strategy for tailored lipid production in oleaginous yeasts.

## Data Availability

The GenBank accession numbers, together with the corresponding species names for all organisms used in this study, are listed as follows: truncated *tesA* from *E. coli* (GenBank accession: QXV67745.1) and *R. toruloides* NP11 *FAS2* (locus tag: RHTO_02139) were retrieved from the National Center for Biotechnology Information (NCBI) database (https://www.ncbi.nlm.nih.gov (accessed on 12 January 2022)).

## Supplementary Materials

The following supporting information can be downloaded at https://www.mdpi.com/article/10.3390/jof12100750/s1. Table S1: Strains used in this study; Table S2: Plasmids used in this study; Table S3: Primers used in this study; Table S4: Comparison of maximum specific growth rate (μ_max_). Figure S1: Construction flowchart of engineered strains. *R*. *toruloides* NP11 was transformed via ATMT to generate Cas9-expressing *Rt*WT; Figure S2: Growth complementation test of *fas2Δ* mutant and ACP domain-engineered *Rt*FAS2 variants complementary strains in *R. toruloides*; Figure S3: Transformant growth phenotypes on cefotaxime-containing YPD plates after ATMT screening; Figure S4: PCR verification and sequencing validation of recombinant strains; Figure S5: Fatty acid composition of *R. toruloides* strains during the logarithmic growth phase in YPD medium; Figure S6: Relative changes in fatty acid composition of *R. toruloides* strains in NL medium.

## Abbreviations

The following abbreviations are used in this manuscript:

ACP	Acyl carrier protein
AS	Acetosyringone
AT	Acetyltransferase
ATMT	*Agrobacterium*-mediated transformation
DCW	Dry cell weight
DH	Dehydratase
ER	Enoyl reductase
FAME	Fatty acid methyl ester
FAS	Fatty acid synthase
FAs	Fatty acids
KR	Ketoacyl reductase
KS	Ketoacyl synthase
LB	Luria–Bertani medium
LCFAs	Long-chain fatty acids
MCFAs	Medium-chain fatty acids
MM	Minimal Medium
MPT	Malonyl/palmitoyl transferase
NL	Nitrogen-limited
PKS	Polyketide synthases
PPT	Phosphopantetheinyl transferase
SD	Synthetic complete
‘TesA	A truncated *Escherichia coli* thioesterase
TFAs	Total fatty acids
YNB	Yeast nitrogen base without amino acids
YPD	Yeast extract, peptone, glucose
