# Supplementary material for: Acyl-Carrier-Protein-Engineering Rewires the Fatty Acid Profile in the Oleaginous Yeast Rhodotorula toruloides
Source: J Fungi (Basel). 2026 Oct 7;12(10):750. doi: 10.3390/jof12100750 (PMC13705844; doi:10.3390/jof12100750)
Supplement: Supplementary file 1 [file jof-12-00750-s001.zip › jof-4592469-supplementary.pdf]

Acyl-Carrier-Protein-Engineering Rewires the Fatty Acid Profile in the Oleaginous Yeast *Rhodotorula toruloides*

Shuang Wang [1,2], Qiongqiong Chen [1,2], Liting Lyu [3] and Zongbao Kent Zhao [1,3,*]

[1] Laboratory of Biotechnology, Dalian Institute of Chemical Physics, Dalian 116023, China; wangs@dicp.ac.cn (S.W.); chenqiongqiong@dicp.ac.cn (Q.C.)
[2] University of Chinese Academy of Sciences, Beijing 100049, China
[3] MOE Key Laboratory of Bio-Intelligent Manufacturing, School of Bioengineering, Dalian University of Technology, Dalian 116024, China; lyult@dlut.edu.cn
* Correspondence: zhaozb@dlut.edu.cn

Supplementary Information

Supplementary Table S1. Strains used in this study.

	Description	Resource
E. coli DH5*α*	*F- λ– endA1 glnV44 thi-1 recA1 relA1 gyrA96 deoR nupG Φ80dlacZΔM15 Δ (lacZYA-argF) U169, hsdR17(rK- mK+)*	Takara
E. coli Fast-T1	*F- φ80(lacZ)ΔM15 ΔlacX74 hsdR(rK- , mK+) ΔrecA1398 endA1 tonA*	Vazyme
Agrobacterium tumefaciens AGL1	*AGL0 recA::bla pTiBo542DT Mop + CbR*	[1]
R. toruloides NP11	*MAT A1*	This lab
NP11-SaCas9-1	*R. toruloides* NP11 with genomic integration of the *SaCas*	[2]
fas2Δ	NP11-SaCas9-1 with genomic integration of the *FAS2Δ*	This lab
*Rt*WT	NP11-SaCas9-1	This lab
*Rt*FAS2	*The fas2Δ* strain with genomic integration of the *FAS2* gene	This study
*Rt*FAS201	*fas2Δ* strain with genomic integration of the *FAS2*(S1026A)	This study
*Rt*FAS202	*fas2Δ* strain with genomic integration of the *FAS2*(S1253A)	This study
*Rt*FAS203	*fas2Δ* strain with genomic integration of the *FAS2*('TesA-ACPII)	This study
*Rt*FAS204	*fas2Δ* strain with genomic integration of the *FAS2*(ACPI-'TesA)	This study

Supplementary Table S2. Plasmids used in this study.

Plasmids	Desccription	Resource
pZPK-SaCas9	pZPK-P_{PGK}-*BLE*-T_{NOS}-P_{GPD}-*SaCas*-T_{HSP}	[2]
pET24b-FAS2	The *FAS2* fragment was inserted into the *Hin*d III/*Not* I site of pET24b (+)	[3]
pUC57-*'tesA*	Amp^R	This study
pZPK-H-MCS	pZPK-P_{PGK}-*HYG*-T_{NOS}-P_{GPD}-MCS-T_{HSP}	Our lab
pZPK-fas2.1	pZPK-P_{GPD}-*HYG*-T_{NOS}-P_{U6b}-*FAS2*-sgRNA	This study
pZPK-MCS	pZPK-P_{GPD}-*MCS*-T_{HSP}, Kan^R	This study
pZPK-FAS2	pZPK-P_{GPD}-*FAS2*-T_{HSP}, Kan^R	This study
pZPK-mACPI	pZPK-P_{GPD}-*FAS2*(S1026A)-T_{HSP}, Kan^R	This study
pZPK-mACPII	pZPK-P_{GPD}-*FAS2*(S1253A)-T_{HSP}, Kan^R	This study
pZPK-'TesA-ACPII	pZPK-P_{GPD}-*FAS2*('TesA-ACPII)-T_{HSP}, Kan^R	This study
pZPK-ACPI-'TesA	pZPK-P_{GPD}-*FAS2*(ACPI-'TesA)-T_{HSP}, Kan^R	This study

Supplementary Table S3. Primers used in this study.

Primers	Sequence (5′-3′)	Description
FAS2-F	CAGATATCCCATGGACATGGTCGCGGCGCAG-GACTTG	Amplify *FAS2* from the plasmid pET24b-FAS2
FAS2-R	GGACTAGTCTAG-TGATGGTGATGGTGGTGCTTCTGGGCGATGAC-GACGGC	
Tnos-U6b-sgRNA-F	GCAGCATGCAAGCTTGGAGCTTGAGCTT-GGGGCGGGATGACCCAGCGCTTTCAC	Amplify *FAS2.1*-sgRNA
FASII.1-sgRNA-R	TAGTAGATTCTGTTTCCAGAGTACTA-AAACCCTTGAACTGGAGCTTGGTGCCAAGAC-CGAAGTCGCTGGAAGAT	
GPD-MCS-RF-R	CTGAATTAACGCCGAATTGAATTCGAGCTCGG-TACCCGGGTCCATGCTGCTGCGATCTGG	Amplify pZPK-H-MCS for MCS vector construction
Thp13-RF-R	TCGGATGCGAGGTGAGAC	
mACP-F	CCCGCGTCGCCCACCCGA	Amplify the mACPI fragment to construct the ACPI inactivation vector
mACPI(S1026A)-R	TGGAGGGTCGCCTTGCCGC	
mACP-F	CCCGCGTCGCCCACCCGA	Amplify the mACPII fragment to construct the pZPK-mACPI vector
mACPII(S1253A)-R	TGCAGGGTCGCCTTGCCGC	
TesA-ACPII-RF-F	CCCAAGGCCGCCCCAG-TCGCCGCCGCCCCTCCCCCTCCCGCTCCTGTCGCTGCCGCGCCTGCCGCCGCCGCTGACACCCTCCTCA	Amplify ‘*tesA* fragment to construct pZPK -‘TesA-ACPII vector
TesA-ACPII -RF-R	ACCGCCAGCTGCGGCCGGAGCGGCGAAC-GCCATCGCGGGGGCAGCAGCAGCAGCTCCAC-CGCCCGAGTCGTGGTTGACGAGG	
ACPI-TesA-RF-F	GGCGGTG-GAGCTGCTGCTGCTGCCCCCGCGATGGCGTTCGCCGCTCCGGCCGCAGCTGGCGGTG-GAGCGCCCGCTGCCGCTGACACCCTCCTC	Amplify the ‘tesA fragment to construct pZPK-ACPI-‘TesA vector
ACPI-TesA-RF-R	TGCTTCTCCTGCATCTT-GTCGAGCTGCTCGGTGTTGATCATAAA-GCCGGCGCCGCCGACAGCCGCGCCGCCTCCGCCTCCCGAGTCGTGGTTGACGAG	
JD-FAS2-F	GAGGCGTTCTGCCGTGTTGTC	Amplify the FAS2 fragment
JD-FAS2-R	CGAACTGGGTGGCGTAGAGGAGA	
JD-mACPs/TesA-F	GGCCCGTCGCCCACTCTCA	Amplify mACPs or ‘*tesA* fragments
JD-mACPs/TesA-R	GTCGGCGAGGCGGTGGCCC	

Supplementary Table S4. Comparison of maximum specific growth rate (μ_{max}).

Strains	*Rt*WT	*Rt*FAS2	*Rt*FAS201	*Rt*FAS202	*Rt*FAS203	*Rt*FAS204
μ_{max} (h^{-1})	0.323 ± 0.004	0.304 ± 0.006	0.314 ± 0.005	0.301 ± 0.004	0.317 ± 0.002	0.345 ± 0.003

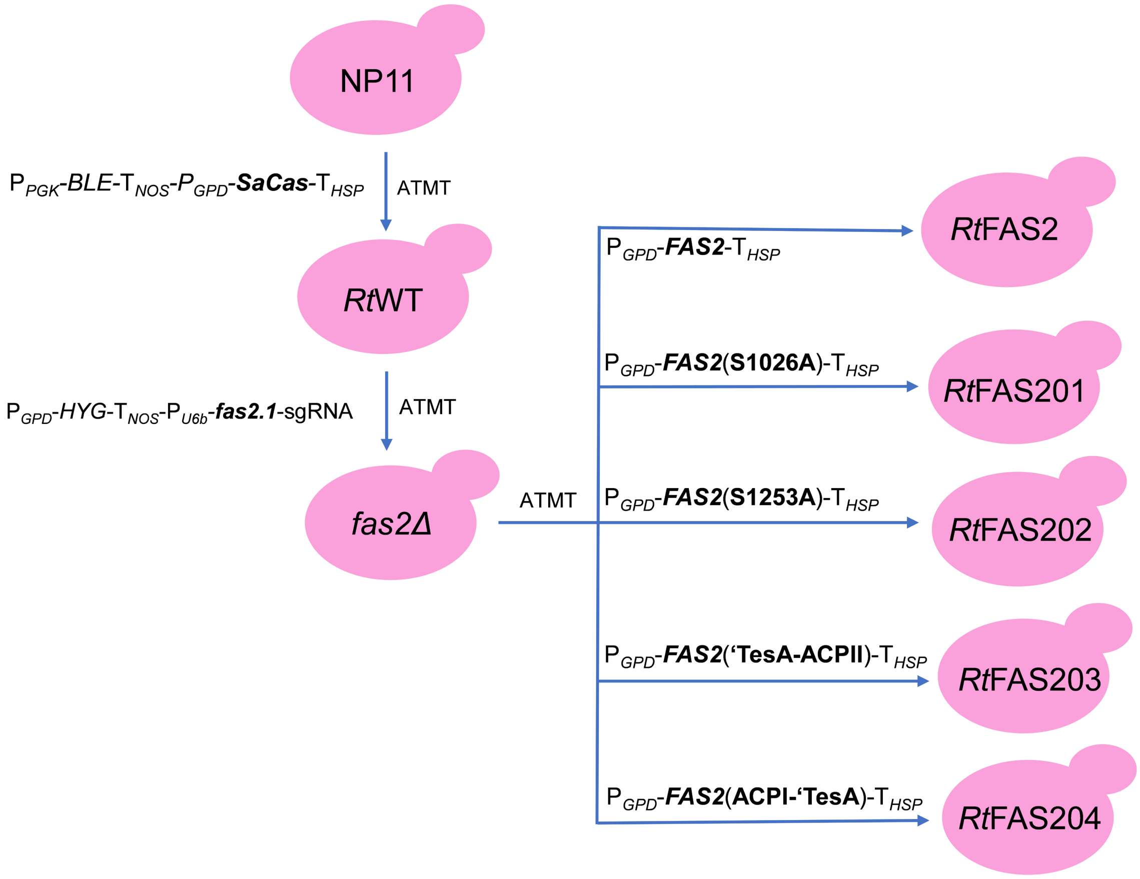

Supplementary Figure S1. Construction flowchart of engineered strains. *R. toruloides* NP11 was transformed via ATMT to generate Cas9-expressing *Rt*WT. CRISPR/Cas9 was further used to delete *fas2* and obtain the *fas2Δ* mutant. Five P_{GPD}-driven FAS2 variant cassettes were individually integrated into the *fas2Δ* mutant by ATMT to construct the complemented strains: wild-type *Rt*FAS2, two ACP point-mutated strains (*Rt*FAS201, *Rt*FAS202), and two *'tesA-ACP* fusion strains (*Rt*FAS203, *Rt*FAS204).

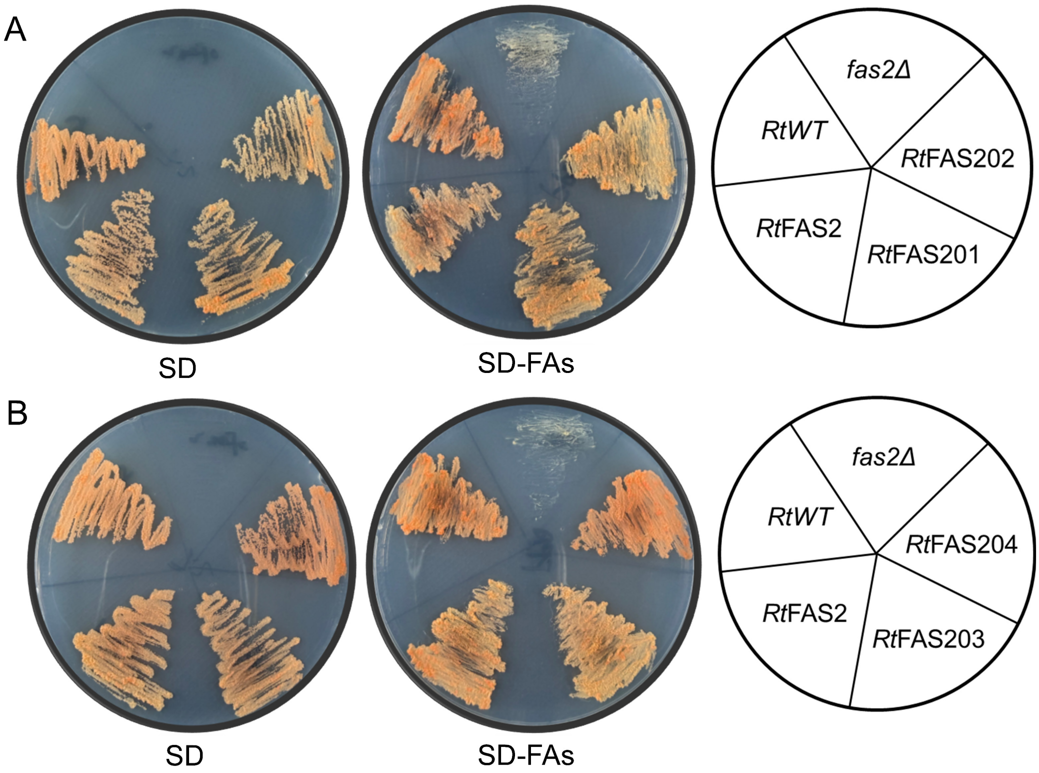

Supplementary Figure S2. Growth complementation test of *fas2Δ* mutant and ACP domain-engineered *Rt*FAS2 variants complementary strains *in R. toruloides*. **(A)** Growth status of *fas2Δ*, *Rt*WT, *Rt*FAS2, *Rt*FAS201 and *Rt*FAS202 streaked on synthetic complete medium without exogenous fatty acids (SD) and SD medium supplemented with fatty acids (SD-FAs). **(B)** Growth performance of *fas2Δ*, *Rt*WT, *Rt*FAS2, *Rt*FAS203 and *Rt*FAS204 on SD and SD-FA solid plates.

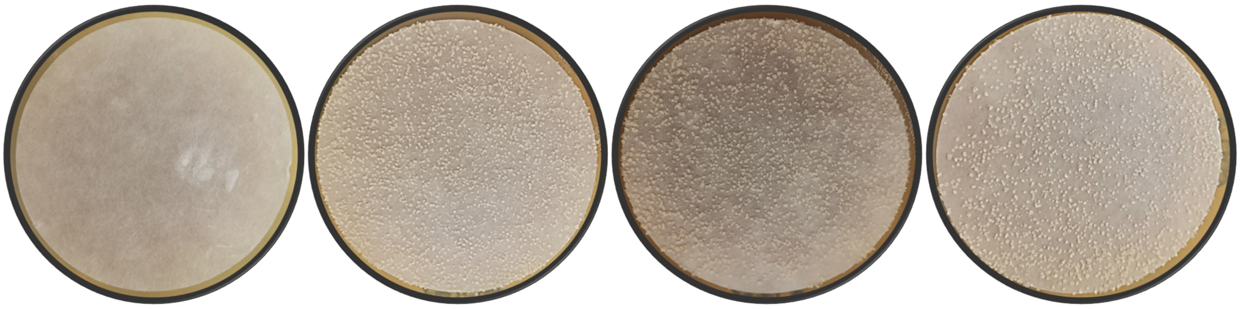

Supplementary Figure S3. Transformant growth phenotypes on cefotaxime-containing YPD plates after ATMT screening. After 2 days of co-cultivation on IM medium, cells were transferred to YPD agar containing 300 μg/mL cefotaxime to eliminate *Agrobacterium* and select positive yeast transformants. From left to right: *fas2Δ*; *Rt*FAS2 (wild-type *Rt*Fas2 control); *Rt*FAS201 (S1026A, mACPI); *Rt*FAS02 (S1253A, mACPII). The *fas2Δ* mutant showed no colony formation. *Rt*FAS2 produced abundant normal-sized colonies. *Rt*FAS201 and *Rt*FAS202 also yielded viable transformants, confirming that single ACP inactivation does not abolish cell viability.

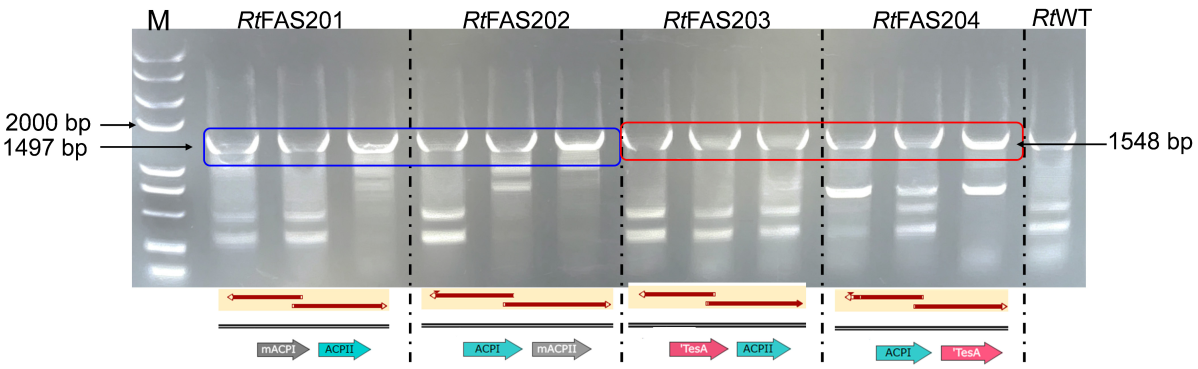

Supplementary Figure S4. PCR verification and sequencing validation of recombinant strains. Upper: Diagnostic PCR agarose gel analysis of the *Rt*FAS201, *Rt*FAS202, *Rt*FAS203, and *Rt*FAS204 recombinants alongside the control strain *Rt*WT (three biological replicates per strain). Lane M: DNA molecular weight ladder, with key size markers (2000 bp) indicated. Strains *Rt*FAS201 and *Rt*FAS202 (ACP domain inactivation) show the expected 1497 bp amplicon, confirming correct genomic integration of the construct. Strains *Rt*FAS203 and *Rt*FAS204 ('TesA-ACP fusion integration) show the expected 1548 bp amplicon, confirming correct genomic integration of the construct. Lower: Schematic representation of the four modified loci validated by Sanger sequencing. Sequencing results confirmed the precise genomic integration of all constructs: the designed point mutations within the ACP domains in RtFAS201 and RtFAS202, and the correct in-frame fusion of the 'tesA sequence with the ACP domain in *Rt*FAS203 and *Rt*FAS204. All engineered strains were constructed in the *fas2Δ* mutant background.

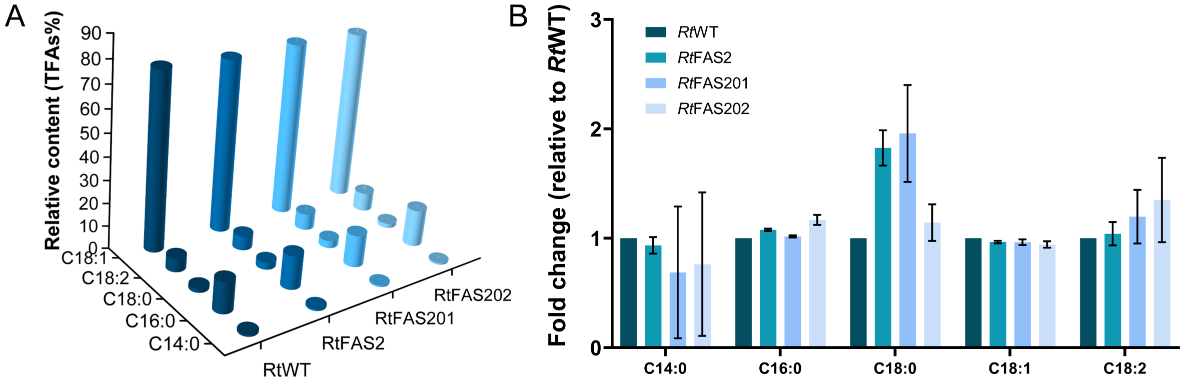

Supplementary Figure S5. Fatty acid composition of *R. toruloides* strains during the logarithmic growth phase in YPD medium. (**A**) Relative content of individual fatty acids (% of total fatty acids, TFAs%) in *Rt*WT, *Rt*FAS2, *Rt*FAS201, and RtFAS202. (**B**) Fold change of each fatty acid relative to *Rt*WT. Values for *Rt*WT were normalized to 1. Data are presented as mean ± SD (n = 3).

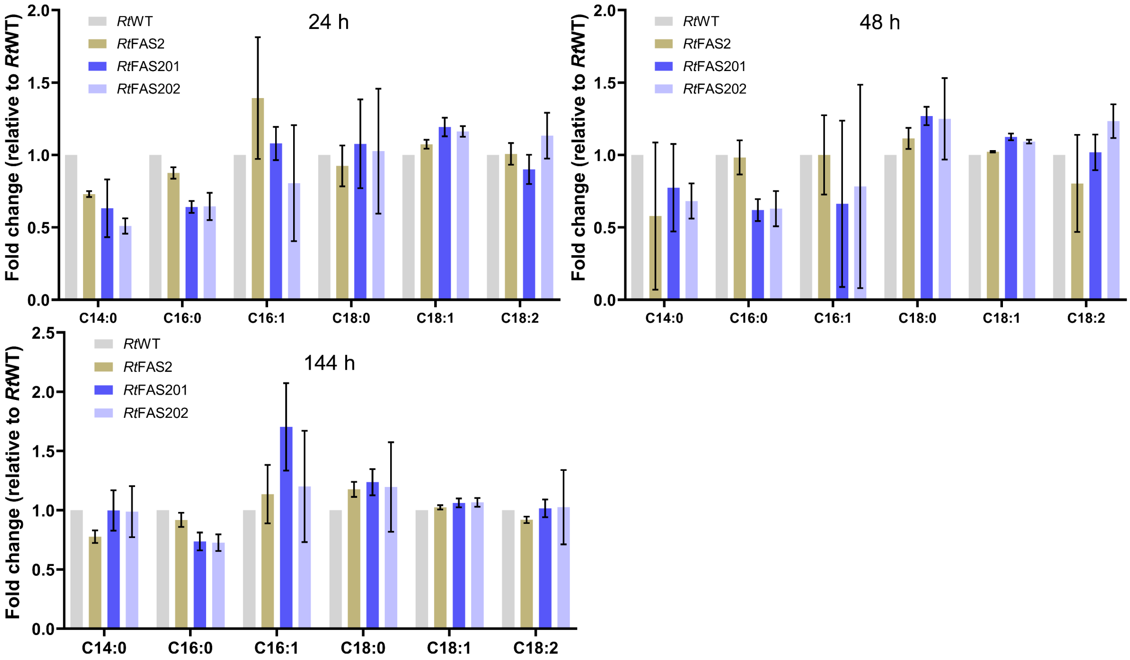

Supplementary Figure S6. Relative changes in fatty acid composition of *R. toruloides* strains in NL medium. Fatty acid profiles were determined at 24 h, 48 h, and 144 h of fermentation for *Rt*WT, *Rt*FAS2, *Rt*FAS201, and *Rt*FAS202. Fold changes of individual fatty acids relative to *Rt*WT are shown; for each time point, *Rt*WT values were normalized to 1. Data are presented as mean ± SD (*n* = 3).

References

1. Lin, X.; Wang, Y.; Zhang, S.; Zhu, Z.; Zhou, Y.J.; Yang, F.; Sun, W.; Wang, X.; Zhao, Z.K. Functional integration of multiple genes into the genome of the oleaginous yeast *Rhodosporidium toruloides*. *FEMS Yeast Res.* **2014**, *14*, 547–555. https://doi.org/10.1111/1567-1364.12140.
2. Jiao, X.; Zhang, Y.; Liu, X.; Zhang, Q.; Zhang, S.; Zhao, Z.K. Developing a CRISPR/Cas9 System for Genome Editing in the Basidiomycetous Yeast *Rhodosporidium toruloides*. *Biotechnol. J.* **2019**, *14*, e1900036. https://doi.org/10.1002/biot.201900036.
3. Zhu, ZW.; Zhang, S.F.; Lin, X.P.; Liu, W.J.; Zhao, Z.K. Expression, purification and characterization of a novel fatty acid synthase from *Rhodosporidium toruloides*. *Chin. J. Biotechnol.* **2014**, *30*, 1414-1423. https://doi.org/10.13345/j.cjb.130626.
